# Development of a Tool for Verifying Leakage Detection in Microfluidic Systems

**DOI:** 10.3390/mi16020124

**Published:** 2025-01-22

**Authors:** Ali Bozorgnezhad, Luke Herbertson, Suvajyoti Guha

**Affiliations:** Division of Applied Mechanics, Office of Science and Engineering Laboratories, Center for Devices and Radiological Health, U.S. Food and Drug Administration, Silver Spring, MD 20993, USA; ali.bozorgnezhad@fda.hhs.gov (A.B.); luke.herbertson@fda.hhs.gov (L.H.)

**Keywords:** microfluidics, leakage testing, medical devices, verification, failure modes

## Abstract

While submissions of microfluidic-based medical devices to the Food and Drug Administration (FDA) have increased in recent years, leakage remains a common but difficult failure mode to detect in microfluidic systems. Here, we have developed a sensitive tool to measure and verify leakages ranging from 0.1% to 10% in leakage detection systems, which can then be used to detect leak in microfluidic devices. Our methodology includes an analytical model that applies hydrodynamic resistance using different fluid-contacting elements (e.g., tubing, junctions, and connectors) to tune the leakage rate based on the application-specific acceptance criteria. We then used three polymer-based microfluidic systems to target leakage rates of approximately 0.1, 1.0, and 10%. The experimental uncertainties in Polyether Ether Ketone (PEEK) tubing were 23.08%, 13.64%, and 1.16%, respectively, while the PEEK-Coated Fused Silica (PEEKsil) tubing system had errors of 0.00%, 0.72%, and 1.59%, respectively, relative to the theoretical values for the same target leak rates. The commonly used commercial grade Cyclic Olefin Copolymer (COC) microfluidic chips produced errors of 7.69% and 5.05%, respectively, for target leakage rates of 0.24% and 1.88%. We anticipate that the proposed bench test method can be useful for device developers as a verification tool for leakage detection systems before assessing flow-mediated leakage failure modes in microfluidic medical devices.

## 1. Introduction

Microfluidic devices have been used in a broad range of applications, including cellular biology, biotechnology, medical devices, and medicine [1,2,3,4,5]. Regardless of the application, a survey conducted [6] by the Microfluidics Association, including early device innovators and device manufacturers, revealed that leaks are one of the most common failure modes encountered in microfluidic devices. The prevalence of leaks in microfluidic devices stems from the large pressure drops and unique fabrication processes. Since the pressure drop in laminar flow is inversely proportional to the fourth power of the diameter, a smaller channel dimension requires a higher inlet pressure, thus increasing the likelihood that the system will leak. Typically, potential device failure modes are expected to be addressed by manufacturers through risk management strategies [7]. However, a more recent analysis conducted by the FDA [8] of adverse events revealed that flow mediated failures (including leakage) are more than twice as likely to occur in microfluidic medical devices compared to larger devices. Therefore, it is important to develop ways to assess leakage accurately for microfluidic medical devices.

A comprehensive review of leakage testing protocols [9] currently used by the medical device industry revealed that there are over 20 standards across 3 major standards organizations that are routinely used in evaluating leaks, most of which are gas-based. The review paper underscored the need for developing simple and standardized protocols for assessing leakage of microfluidic devices using physiologically relevant liquids (e.g., water, saline, blood) to better mimic the intended use scenario.

A complicating aspect in microfluidics is the difficulty in accurately measuring low flows on the microscale. Recent hydrodynamic resistance measurements of simple straight channels revealed that the error associated with flow sensors is often within ±10%, but it may exceed 50% in some cases [10]. This large uncertainty in flow measurements has potentially led researchers to propose alternate methods [11] of flow measurement in the micro-flow regime. Of these flow measurement techniques, the most reliable may involve gravimetric measurements. In fact, manufacturers often use gravimetric methods to assess the accuracy of low-flow devices due to the measurement sensitivity even at low flows [12,13].

The allowable or acceptable amount of leakage, i.e., fluid loss, often depends on the application [14] or the clinical risk associated with the leak. For some applications, small leaks may delay patients from receiving life-saving therapy and result in device recalls [15,16]. In other cases, a device leak may not negatively impact performance or the ability to diagnose a disease, but it may still be hazardous to the device user. Nevertheless, there is a need to develop a general protocol for quantifying and verifying leakage rates for a variety of biomedical applications.

Several leakage detection systems have been commercially developed [17,18,19,20,21], but, to the best of our knowledge none of these systems have specifically been built to quantify leakage in microfluidic devices. Therefore, verifying the leakage of these commercial (or non-commercial) systems prior to their use in characterizing medical device leakage is necessary. Currently there are no standardized protocols for verifying leakage systems. The objective of this paper is to develop a simple tunable leakage verification methodology that can be used to verify leakage in any commercial or non-commercial leakage detection systems. This methodology is tunable, as it can provide a multitude of target leakage rates (e.g., 0.1%, 1.0% etc.) that would be acceptable for the specific intended use of the microfluidic medical device. Following the use of this protocol to verify the ability of commercial/non-commercial leakage detection systems to measure the acceptable target leakage, the leakage detection system can then be used to characterize the leakage rate of the specific microfluidic medical device (independent of its application).

## 2. Materials and Methods

We investigated several different polymers commonly used in microfluidics to offer manufacturers with various material choices for developing their verification leakage test system (Table 1). Note that since the leakage verification is intended to be used to verify leakage detection systems and not the microfluidic medical devices themselves, and the leakage detection systems are typically expected to be device- and material-agnostic, the material used to build the leak verification system described here does not influence the material selection of the medical device. All the experiments were performed in triplicates. We chose PEEK and PEEKsil over other available microfluidic tubing materials because of their ability to tolerate high pressure (>7000 psi) and their negligible change in internal diameter due to fluid pressure. Since permissible leakage in a device is application-specific, and there is no consensus on what target leakage rates are acceptable, we chose to study a clinically relevant range of leakage rates that span two orders of magnitude.

**Figure 1:**
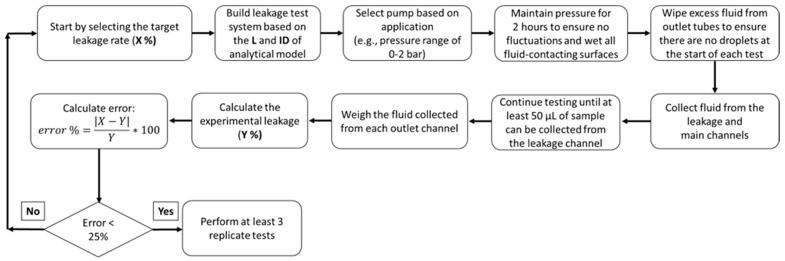
Flow diagram showing the methodology used to obtain the target leakage with the PEEK leakage test system. First, the target analytical leakage (%) was selected, and then the tubing L and ID were determined. Then, the test was performed, and replicate measurements were made only when the error was within the acceptable margin of 25%.

The flow diagram for designing and testing PEEK and PEEKsil tubing is shown in Figure 1. The calculations are provided in the Supporting Information (Section S1). Our approach can be customized for achieving a different target leakage rate beyond the expected range of 0.1–10% presented in this article.

Figure 1 provides the iterative methodology of obtaining the target leakage. An Excel^®^ (Microsoft, Redmond, WA, USA) spreadsheet (Supporting Information Spreadsheet S1) is also provided so that manufacturers can determine which parameters need to be adjusted to come up with a target leakage rate. The spreadsheet inputs are fluid viscosity, length (L), and internal diameters (ID) of the tubing, connectors, and T-junction. Note that the viscosity is needed for calculating hydrodynamic resistance and it does not affect the leakage rate calculations. While we planned to achieve target leakages of 0.1, 1, and 10% with the PEEK and PEEKsil test systems, the actual values of the target leakage rates obtained (Table 1) were slightly different because of the tubing L and ID that were used. However, a manufacturer can iteratively change these geometric variables to obtain a target leakage percentage specific to their application. In this example, these calculations and experiments were performed iteratively until the relative error of the target leakage (i.e., analytical prediction) and the experiments were less than 25%. This error criterion provides flexibility for differences from the target leakage because of the commercial parts that are used to build the test system.

**Figure 2:**
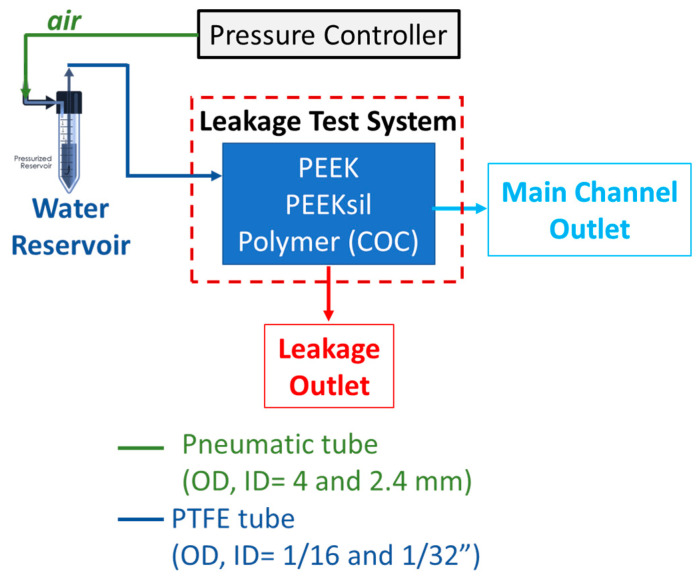
Experimental setup that was used for measuring the leakage rate. The fluid mass at the main channel and leakage channel outlets were measured gravimetrically. A constant pressure of air was used throughout the experiment.

Figure 2 shows the experimental leakage test system that was used to obtain various leakage rates. The temperature and humidity were 23 ± 2 °C and 55 ± 5%, respectively. Note that the relative humidity of the room can be monitored for long experiments to make sure that the evaporation is minimized. All components, including the pressure controller, test media (water), and microfluidic leakage test system, were placed in the room to equilibrate for several hours before testing. Electrically powered components, such as pressure controller, were powered on for at least 30 min before testing. The pressure controller required filling a reservoir (i.e., pressure pump) at least 30 min before initiating measurements. Before starting any experiments, the entire fluidic system underwent purging which involved flowing water through the system below the maximum allowable pressure of the leakage test system. Here, water was pumped through the system using the ElveFlow OB-1 pressure controller system (ElveFlow, Paris, France) with an output pressure range of 0 to 2 bar. The pressure stability was 0.1 mbar, and the pressure response and settling time (the time required to reach 95% of the set point) were 10 and 50 ms, respectively. The pressure controller was connected to a 2.5-bar compressed air line in conjunction with an in-line pressure regulator, dehumidifier (humid trap), and a 5 µm particle filter in series. The pressure controller position was placed at a higher elevation than the pressurized tank and leakage test system and a hydrophobic disk filter was used to reduce the potential of liquid backflow into the pressure controller. The pressure controller was factory calibrated and subsequently recalibrated onsite every three months. After each replicate test run, the pressure source was deactivated or shut down. The pressure controller was operated within a clean, dry, well-ventilated setting, maintaining a humidity level of no more than 60%.

A constant air pressure was applied to the system, but the fluid flow rate drifted over the duration of each test. Before starting the experiments, the main channel was wetted. Next, the main outlet was blocked using a plug, causing all fluid to flow through the leakage channel to wet all fluid-contacting surfaces. Depending on the target leakage rate and the inlet flow rate to the test system, it took approximately 1 to 8 hours to fully wet the leakage channel. After all the surfaces of the channels and tubing were wetted, the plug through the leakage outlet was removed. The outlet PTFE tubes of the main and leakage channels were positioned at the bottom of separate collection glass vials and plastic Petri dishes, respectively, to measure the fluid mass at each outlet. Data collection was initiated after ensuring that the system was free of unwanted air bubbles and there were no droplets at the end of the main outlet and leakage channels. Sample collection occurred until at least 50 µL of water was collected at the leakage outlet by marking a line on the collecting glass vial that corresponds to the 50 µL mark. Gravimetric measurements for the outlet and leakage channels were then made. This volume was chosen so that the corresponding weight (50 mg) was more than three orders of magnitude larger than the scale’s lowest limit of detection.

Currently, most commercial thermal microfluidic flow sensors have an intrinsic error of 5–10%, which is not suitable for measuring small leaks of 0.1 to 10% in microfluidics. Specifically, the signal-to-noise ratio is not high enough for certain applications to accurately measure leakage based on the percentage of the main inlet flow that does not flow to the main outlet. To offset this shortcoming, the leakage percentage calculations were measured gravimetrically using a calibrated scale with a minimum weight of 0.01 mg, a precision of 0.01 mg, and maximum weight of 81 g (Ohaus Discovery DV215CD, OHAUS Corporation, Parsippany, NJ, USA). The weight of the empty vial and Petri dish for the leakage and the main channel outlets were accounted for and zeroed before the start of each experiment. The leakage percentage was calculated using Equation (1) below. The Petri dish and the vial were measured empty initially ($w_{initial}$) and once again after end of the experiments ($w_{final}$) to obtain the corresponding initial and final weights. For simplicity, we used a single scale and measured the weights sequentially: the main outlet flow collected in a Petri dish was measured, followed by the leakage outlet, which was collected in a vial.

(1) $$leakage=\frac{{\left[w_{final}-w_{initial}\right]}_{leakage\;outlet}}{\left({\left[w_{final}-w_{initial}\right]}_{main\;outlet}+{\left[w_{final}-w_{initial}\right]}_{leakage\;outlet}\right)}*100$$

Because the numerator and denominator of Equation (1) were rounded to the nearest 0.01 mg, the leakage values were rounded to the nearest 0.01 mg. Thus, the minimum leakage percentage measurable using our scale is 0.01%.

Ultrapure water was used in the experiments at 23 °C. The flow was assumed to be incompressible and laminar.

**Figure 3:**
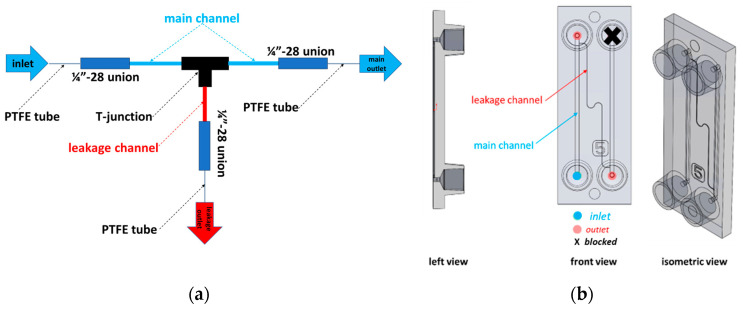
(**a**) PEEK and PEEKsil leakage test systems. Most of the fluid flows through the main outlet channel and only a small portion goes through the leakage channel. Table S1 provides details on the dimensions of the tubing used. (**b**) The COC chips are shown in three different views.

The outer diameter (OD) of all tubing, including Polytetrafluoroethylene (PTFE), PEEK, and PEEKsil tubes, was 1/16”. The PEEK and PEEKsil leakage test systems were used with 1/4”-28 compression fittings for connecting with tubing unions and T-junctions, as shown in Figure 3a. The details of the dimensions of the leakage and main channels, including their IDs and Ls, are provided in Table S1. The main and leakage PEEK and PEEKsil tubes were directly connected to the T-junction. The T-junction was a PEEK tee (0.02” thru hole, F-300, IDEX P-727), and the fluid union was made of Ethylene Tetrafluoroethylene (ETFE) with a thru hole of 0.03” using 1/4”-28 flat-bottom threading ports. The COC polymer chips had straight leakage channels with cross-sectional dimensions of 50 × 50 and 100 × 100 μm (Figure 3b). The inlet and outlet of the chips were connected to 1/32” ID PTFE tubing using mini-Luer connectors. The PTFE tubing containing water from the fluid tank to the system was maintained in a diagonal position to prevent and remove any potential air bubbles within the test system. While this test setup creates a pressure head, the leakage calculations are independent of this hydrostatic pressure change. The contributions of the tubing, connectors, and test systems, as well as the corresponding hydrodynamic resistance calculations, to the leakage rate are provided in the Supporting Information.

The error is defined as the relative deviation of the target or predicted leakage percentage from the average experimental values. This error was calculated using the following equation, where the number of replicates varied from a total of 3 (for 1 PEEKsil leakage test system tested in triplicate) to 9 (for 3 PEEK leakage test systems, each tested in triplicate). Fundamentally, this error measures the deviation of the experimental obtained values from the target leakage, i.e., the analytically obtained target leakage as a percentage.

(2) $$error\;\%=\frac{\left|{average\;leakage}_{experimental}-{leakage}_{target}\right|}{{average\;leakage}_{experimental}}*100$$

Here, “${average\;leakage}_{experimental}$” in Equation (2) is the average of all replicates and designs for a specific target leakage. Since averaging may have a nullifying effect on the error, we also determined the root mean squared error (RMSE) to compare each experimental data point separately with the target leakage using equation below:

(3) $$RMSE\;\%=\sqrt{\frac{\sum_{i=1}^{n}{\left({{leakage}_{experimental}}_{i}-{leakage}_{target}\right)}^{2}}{n}}$$

where n = 3 or 9 for PEEKsil and PEEK, respectively. Here, “${{leakage}_{experimental}}_{i}$” in Equation (3) is the value of the experimental leakage calculated specifically for the replicate i.

Note that our test setup did not have the feedback loop (due to logistical challenges) to control the flow rate, and the flow rate was varied during experiments. While it is not necessary to have a constant flow rate to achieve a specific leakage percentage (refer to Equation S6 in the Supporting Information, which is independent of flow rate and relies only on the channel dimensions), device developers may consider using an in-line flow sensor with feedback loop to the pressure controller to achieve and maintain their desired flow rate.

## 3. Results

**Figure 4:**
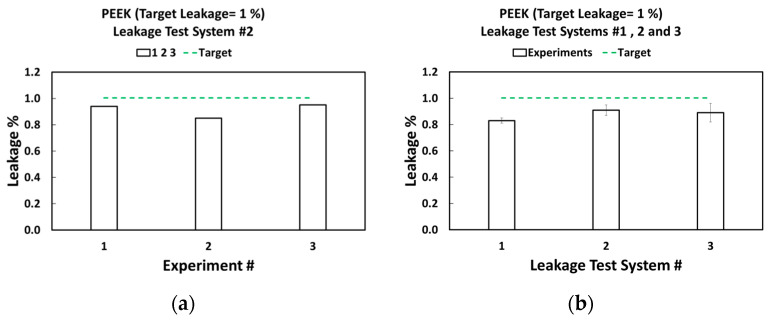
(**a**) Run-to-run variability for PEEK at a target leakage of 1%. (**b**) Leakage-test-system to leakage-test-system variability for PEEK at a target leakage rate of 1%. The error bars are from the experiments performed in triplicates.

The results of the triplicate experiments for PEEK leakage test system #2, targeting a leakage of 1%, are shown in Figure 4a. The 1% leakage serves an example of the data; additional results for other leakage percentages and leakage test systems can be found in the Supplementary Data. The experimental values for 1% leakage across multiple test units align closely with the target values, resulting in an average error of 9.89%. Additionally, the variability for a single leakage test system tends to be low (i.e., leakage test system # 2 had an error of just 0.04%). The results of the triplicate experiments for each PEEK leakage test system (n = 3) targeting a 1% leakage are presented in Figure 4. The average leakage percentage across each individual leakage test system is 0.88%, resulting in an error of 13.64% compared to the target leakage rate of 1%. The aggregated standard deviation for these experiments (n = 9) is 0.06%, further confirming the low variability in leakage percentages across different leakage test systems and repeats.

The experimental leakage compared to the target leakage for all the PEEK leakage test systems is illustrated in Figure S3a. The target leakages were set at 0.1%, 1%, and 10.47%, with corresponding average leakages of 0.13%, 0.88%, and 10.35%. This resulted in errors of 23.08 ± 0.00%, 13.64 ± 0.06%, and 1.16 ± 0.94%, respectively.

Similarly, Figure S3b shows the experimental leakage in relation to the target leakage for the PEEKsil leakage test systems, which have target leakages of 0.09%, 1.38%, and 11.47%. The average experiment leakages for these targets were 0.09%, 1.39%, and 11.29%, resulting in errors of 0.00 ± 0.00%, 0.72 ± 0.13%, and 1.59 ± 0.03%, respectively. Note that because of the precision of the scale used, and the concomitant rounding off average leakage, standard deviations, and errors, all the lowest target leakage experiments resulted in 0.00%.

The experimental leakage of the COC chips, compared to the target leakage, is shown in Figure S3c. The results in errors were 7.69 ± 0.02% and 5.05 ± 0.11%, respectively. We were not able to calculate the target measurement of 10% because it was designed by a third party and their design did not include a chip for 10%.

**Figure 5:**
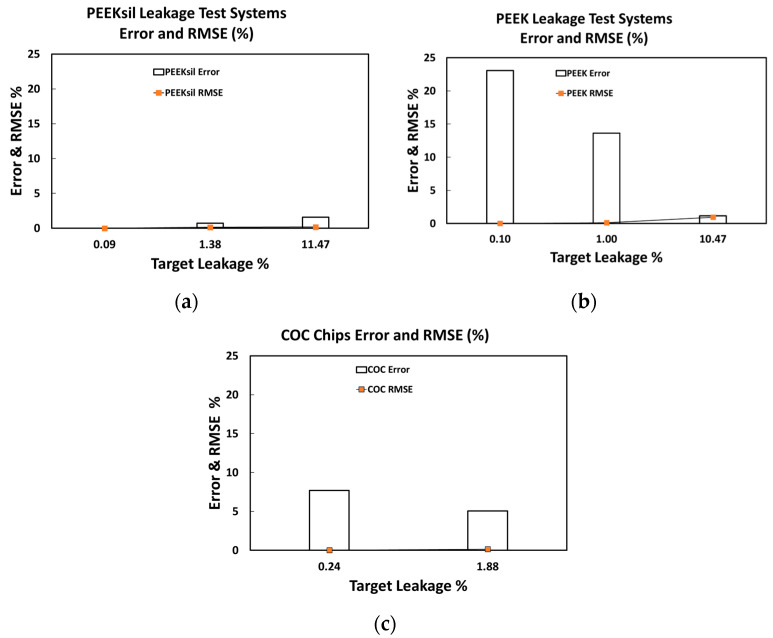
Error and RMSE of the (**a**) PEEK, (**b**) PEEKsil, and (**c**) COC leakage test systems.

The error and RMSE of the PEEK and PEEKsil leakage test systems are shown in Figure 5. The error was much lower for the target leakages of 0.1 and 1% in the PEEKsil leakage test system compared to the PEEK system, while the error for the target leakage of 10% was similar, at around 1%, for both the PEEK and PEEKsil leakage test systems.

## 4. Discussion

Flow-related failures in microfluidic medical devices, particularly bubbles and leaks, have hindered otherwise promising microfluidic technologies. However, as microfluidic technologies mature toward increasing adoption in biomedical applications, it is important to develop methodologies to characterize and mitigate failure modes. Unfortunately, despite the community identifying needs [23] to develop standardized methodologies in areas such as flow control and interconnections, progress in these areas remains slow [24]. Therefore, there is an immediate need to develop methodologies to characterize failure modes, including leakage, in microfluidic medical devices.

For high-risk medical devices [25], including those that are implanted, manufacturers often have in-line quality control for leaks or mechanical integrity of their devices. However, there are also lower- or moderate-risk devices, for which the risk of patient harm from leakage may be less but still impactful. In fact, prior analysis [8] of device submission trends revealed that most microfluidic medical devices are expected to fall in the moderate risk category. For some of these devices, the manufacturers may need to define an acceptable amount of leakage in their device and then perform verification testing on their leakage system before assessing leakage rate in their device leak. However, currently, there is a lack of simple leakage-testing methodologies for microfluidics [9], and, to our knowledge, there is no verification test protocol available for characterizing the sensitivity of microfluidic leakage systems. Our proposed approach can potentially bridge the regulatory science gap.

First, device developers can use the supplemental Excel^®^ spreadsheet (provided in Supporting Information Spreadsheet S1) to tune their own target leakage values for testing and then verify the leakage of their test system using a PEEK and PEEKsil leakage test system before evaluating their own device for safety. We anticipate that early device developers who rely on visual techniques for leakage assessment may benefit from following the proposed quantitative approach to characterize the leakage in their microfluidic leakage test systems before their proof-of-principle devices are scaled up, undergo rigorous verification and validation testing, and receive FDA clearance or approval to become legally marketed medical devices. For this scenario, developers can first use our methodology to verify that they can accurately measure a target leakage value before moving onto quantifying the leakage in their finished devices. When planning to use this approach, it may be beneficial to consider the following:

- Use a commercial leakage system or develop a hand-built leakage system to characterize the leakage rate of the developed microfluidic application. Then, the developer can assess if that leakage rate is acceptable from a clinical standpoint and terms of risk to the patient. If not, then the design would need further modification. However, if the leakage rate is acceptable, then proceed to build a verification leakage system as described below.
- Developers can input values of L and ID for the PEEK or PEEKSil leakage test systems, the connecting tubes, and the T-junctions in the Excel spreadsheet. It may be easiest to keep the physical leakage test setup the same as described in Materials and Methods section above. The target leakage is most sensitive to the ID of the leakage channel tubing because hydrodynamic resistance scales as the inverse of the fourth power of the ID. For example, an increase in ID of the leakage tubing from 100 µm to 120 µm causes the target leakage rate to increase from approximately 0.1 to 0.2%.
- After determining the L and ID, the developer can purchase the tubing with the necessary ID and cut it to the desired lengths. When choosing between different materials, such as PEEK and PEEKsil, the developer may need to carefully consider the cost versus the likely experimental error. PEEK is less expensive than PEEKsil and can be easily cut to the desired lengths in a laboratory. The proposed PEEKsil leakage test system appeared to have lower error (<2%) compared to the PEEK leakage test systems, but it is often purchased in precut lengths. This reduced error can be reconciled with the tolerance of the ID of the PEEK and PEEKSil leakage test systems. The PEEKsil tubing is often pre-cut (using electrochemical machining), and the ID tolerances appear to vary by 2–6% [26]. Comparatively, PEEK tubing has an ID tolerance that remains constant around 25 µm when the tubing ID ranges from 50 µm to 1000 µm. This implies that the ID tolerance can be as much as 50% at the smallest ID and gradually decreases to about 3% at larger diameters (PEEKsil [26], PEEK [27]). Due to the strong dependence of hydrodynamic resistance on ID, the larger tolerance in PEEK leakage test systems combined with manufacturing variability likely contributes to the higher error (23.08%) in our 0.1% leakage verification system. Therefore, choosing PEEKsil over PEEK may be desirable if a leakage verification system with lower associated errors is desirable.
- The advantage of the potentially lower error with PEEKsil may not be as pronounced for higher target leakage rates. At the target leakage of 10%, both the PEEK and PEEKsil leakage test systems demonstrated low error rates (Figure 5a,b), implying that the PEEK leakage test system may be more suitable and cost-effective for larger leakage applications of >10%. Note that since the system-to-system variability was not tested with PEEKsil, the errors associated with multiple test systems are to be interpreted with caution.

An additional benefit to the methodology described here is the lack of reliance on thermal microfluidic flow sensors. As the precision of these sensors is typically expected to be 5–10%, they may not be sensitive enough to detect very small leakages (e.g., 0.1%). Hence, gravimetric measurements or relying on Coriolis flow technology may be more appropriate. Additionally, note that since the leakage rate (Equations (S2)–(S6) in the Supporting Information) is independent of the fluid used, its viscosity, and the viscosity’s dependence on temperature, our methodology can be used for other fluids at other physiologically relevant temperatures as well. A third aspect to note is that our leakage verification system is completely independent and removed from the material used to develop the microfluidic medical device.

A second use of our tool may also be to develop standardized leakage test systems to perform inter-laboratory studies before developing standards that can be more widely adopted in biomedical and chemical applications. Such an effort is currently underway [22,28]. To eliminate any potential effect of batch-to-batch variability that may be possible in PEEK and PEEKSil leakage test systems, it may be meaningful to use injection-molded COC chips as they appear to be the preferred choice in biomedical applications [8]. Given that the hydrodynamic resistance offered by other tubing materials and junctions can be comparable to that of the main channel of the COC chips, the dimensions of these tubing sets and junctions may need to be made consistent across the laboratories before taking any steps to standardize leakage testing methodologies.

Our methodology would need further modification to be used by a device developer to directly measure leakage in their microfluidic device, i.e., as a leakage detection system. An actual device will not have a direct leakage outlet (Figure 2 like our polymer systems. The leakage in a microfluidic device is likely to occur at undetermined locations within the device. In this scenario, and contrary to how we used a pressure-driven inlet system (Figure 2), the developer would need to have a means to measure the inlet flow rate. They would also need to measure the outlet flow rate as we did. Measuring both the inlet and outlet flow rates would then enable a developer to determine the leakage flow rate for their device. While developing this type of leakage detection system, it may be beneficial to use gravimetric means to measure the flow rates, since the error associated with the microfluidic flow sensors may exceed the sensitivity required to measure small leakage rates of 0.1–1%. Additionally, the developer would also need to be cognizant of evaporative losses that may occur, which would be highly specific to the laboratory conditions.

Limitations:

- Since we performed our experiments at ambient pressure, we assumed that the flow was incompressible. Our calculations showed that the Reynolds number remained below 100, hence the flow was assumed to be laminar.
- Our findings are limited to Poiseuille flow in tubing and connectors with circular cross-sectional areas that remain constant throughout their length. Also, water was used at 23 °C with a dynamic viscosity of 0.001 Pa·s, which is a Newtonian fluid. Therefore, our approach may not be appropriate for studying applications involving non-Newtonian fluids (e.g., blood).
- In calculating the errors, we were largely limited by the sensitivity of our gravimetric method, which had a resolution to the nearest 0.01%.
- Unlike the PEEK test system experiments that were conducted on three separate units, only one PEEKsil test system was used to evaluate leakage.
- We did not have any design input to the achievable target leakage rates using the COC chips.
- It is important that all junctions and connectors, except for the leakage outlet channel, remain leak-free.
- We did not investigate the impact of surface functionalization on the PEEK and PEEKsil tubes.
- Our methodology was validated only for verifying leakage detection systems and was not validated for use as a leakage detection system.

## 5. Takeaways for the Microfluidic Medical Device Manufacturers

- Device developers can verify the leakage of their setup using our leakage system at approximately 0.1, 1.0, and 10% leakage rates. Developers can achieve these leakage percentages if they use connectors, T-junction, unions, and tubing with the exact same dimensions and geometry under the conditions described in this article. Note that our methodology is independent of the inlet pressure and inlet flow rate. Hence, developers can use our methodology with pressure and flow rate conditions that are applicable for their device use scenarios. Any change in the dimensions and geometry of fluidic elements, however, will change the target leakage percentage, and developers can see the effect of the changes using the Excel spreadsheet.
- For developers who want to use different target leakage rates, they can use the Excel^®^ spreadsheet to select a target leakage specific to their application. The needed lengths and internal diameters of PEEK and PEEKsil leakage test systems can be determined using the spreadsheet to predict any target leakage rate based on the assumptions described above. We used the PEEK and PEEKsil over other available microfluidic tubing materials as surrogates of the microfluidic chip because of their price and availability, ability to tolerate high pressure, and negligible change in internal diameter due to the fluid pressure.
- PEEK tubing can be easily used to construct a leakage test system for verification at a cheaper price (per unit feet) compared to PEEKsil.
- PEEKsil appears to also be an alternative option to PEEK, as it offers the lowest error in measuring leakage rate at a reasonable price. Thus, PEEKsil may potentially reduce the number of iterations a manufacturer may need to reach their desired target leakage rate for developing a leakage test system.
- The COC chips, despite being significantly more expensive (including the cost of making a specific mold), do not appear to offer significantly lower errors compared to the PEEK and PEEKSil leakage test systems.

## Figures and Tables

**Table 1:** General overview of the target leakages, materials used in the leakage test system, and replicate information. The $, $$, and $$$ correspond to the values ~$10, ~$100, and >$1000 (including the price required for making a mold), respectively. These prices are approximations, as the actual price may depend on number of chips, customization, etc. Additionally, the pricing estimates exclude costs associated with the rest of the leakage test system (e.g., tubing). The COC chips were provided by a collaborator [22].

Target Leakage %	Leakage Test System	Number of Designs	Price
0.1	Polyether Ether Ketone (PEEK) tubing (IDEX Health & Science, Oak Harbor, WA, USA)	3	$
1			
10.47			
0.09	PEEK Coated Fused Silica (PEEKsil) tubing (Trajan Scientific and Medical, Ringwood, Australia)	1	$$
1.38			
11.47			
0.24	Cyclic Olefin Copolymer (COC) chip (microfluidic ChipShop, Jena, Germany)	3	$$$
1.88			

## Data Availability

The data reported in this article is provided here.

## Supplementary Materials

The following supporting information can be downloaded at https://www.mdpi.com/article/10.3390/mi16020124/s1: Figure S1: Schematic of the analytical model for the PEEK and PEEKsil leakage test system; Table S1: General overview of the target leakages, materials and dimensions used in the leakage test system.; Figure S2: Schematic of the analytical model for the COC chip. Figure S3: Average experimental and target leakages: (a) All PEEK leakage test systems, and each bar represents three leakage test systems tested in triplicate experiments (n = 3); (b) All PEEKsil leakage test systems, and each bar is one leakage test system tested in triplicate experiments (n = 3); (c) COC Polymer chips with 50 × 50 µm and 100 × 100 µm leakage channels, with target leakage per-centages of 0.24 and 1.88, respectively, and each bar represents three leakage test systems tested in triplicate experiments (n = 3). Spreadsheet S1: Calculations to Determine Target Leakage; Spreadsheet S2: Data File with Raw Data and Calculations.
