# Supplementary material for: Development of a Tool for Verifying Leakage Detection in Microfluidic Systems
Source: Micromachines (Basel). 2025 Jan 22;16(2):124. doi: 10.3390/mi16020124 (PMC11857335; doi:10.3390/mi16020124)
Supplement: Supplementary file 1 [file micromachines-16-00124-s001.zip › Supplementary Materials.docx]

Supplementary Materials

Development of a Tool for Verifying Leakage Detection in Microfluidic Systems

Ali Bozorgnezhad, Luke Herbertson and Suvajyoti Guha *

Division of Applied Mechanics, Office of Science and Engineering Laboratories, Center for Devices and
Radiological Health, U.S. Food and Drug Administration, Silver Spring, MD 20993, USA

***** Correspondence: suvajyoti.guha@fda.hhs.gov

**S1. Analytical Model to Calculate Leakage**

An analytical model was developed to predict the target leakage using the hydrodynamic resistance of the fluidic elements defined by the following equation, where the L and D_h_ are the length and hydrodynamic diameter of the fluidic element (e.g. tubing, connector, or junctions), respectively.

| $Hydrodynamic Resistance=R=\frac{pressure drop}{flow rate}=\frac{\Delta P}{Q}\sim\frac{L}{{D_{h}}^{4}}$ | (SE. 1) |
| --- | --- |

The hydrodynamic diameter of the tubing with circular cross-section are their internal diameter (I.D). The hydrodynamic resistance of any tubing is calculated using the following equation, where µ, L and D are the fluid viscosity, length and ID of the element, respectively:

| $R_{circular}=\frac{128\mu L}{\pi D^{4}}$ | (SE. 2) |
| --- | --- |

The hydrodynamics resistance of the fluidic elements with rectangular cross-sections are calculated using the following equation, where the µ, L, h and W are the fluid viscosity, length of element and height and width of the element, respectively:

| $R_{rectangular}=\frac{12 \mu L}{1-0,63\times\left( \frac{h}{W} \right)}\left( \frac{1}{h^{3}W} \right)$ | (SE. 3) |
| --- | --- |

The schematic of the analytical model for the PEEK and PEEKsil leakage test systems is shown in Figure S1. The cross-sections of all the PEEK and PEEKsil leakage test systems fluidic elements were circular.


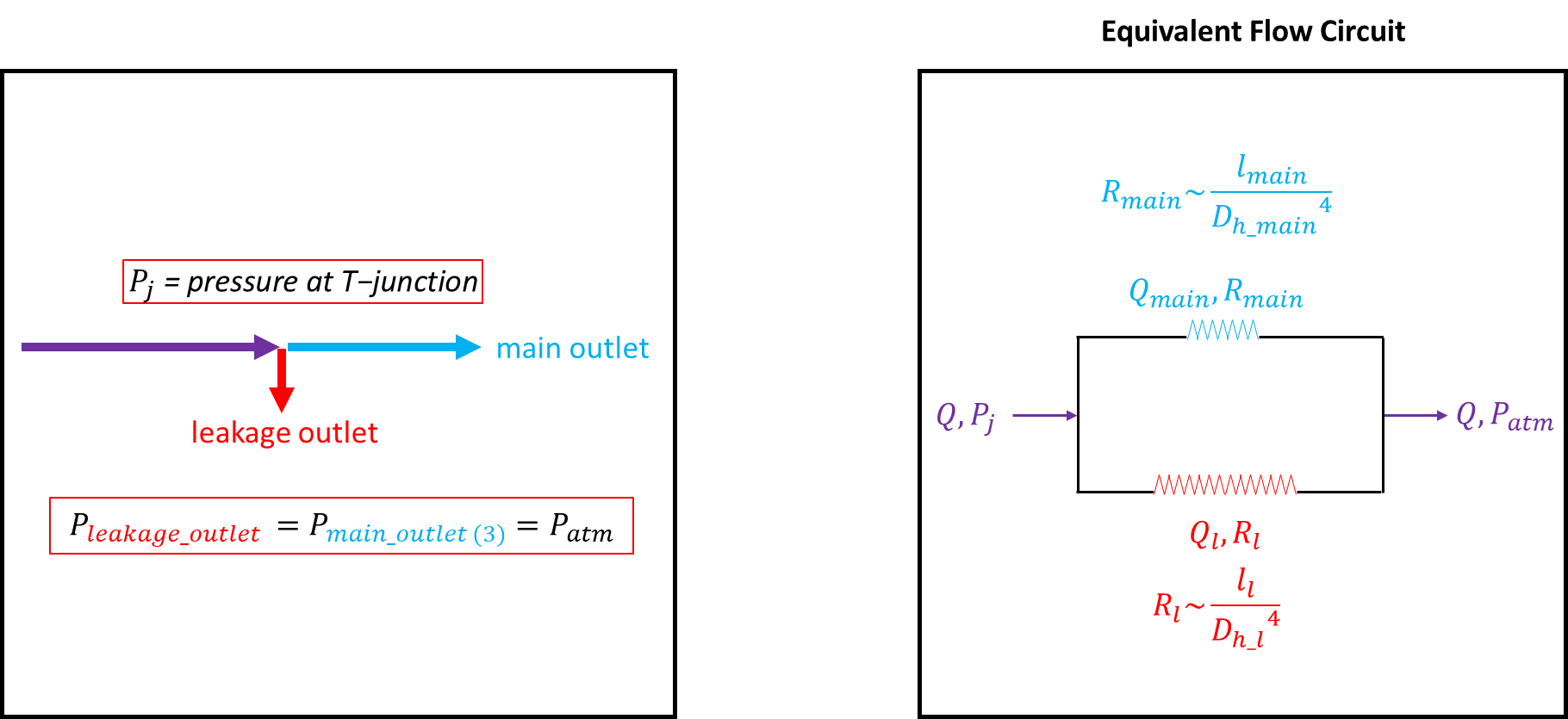


**Figure S1.** Schematic of the analytical model for the PEEK and PEEKsil leakage test system.

| and  $Q_{l}=\frac{P_{j}-P_{atm}}{R_{l}}$  $Q_{main}=\frac{P_{j}-P_{atm}}{R_{main}}$ | (SE. 4) |
| --- | --- |

Q_main_ and R_main_ and Q_l_ and R_l_ are the flow rates and hydrodynamic resistance of the main and leakage channel outlets, respectively. The P_j_ and P_atm_ are the pressure at the T-junction and the ambient pressure, respectively. The above equations when expressed as a ratio become -

| $\frac{Q_{l}}{Q}=\frac{Q_{l}}{Q_{main}+Q_{l}}=\frac{R_{main}}{R_{l}\text{ + }R_{main}}=\left( \frac{1}{1\text{+}\frac{R_{l}}{R_{main}}} \right)$ | (SE. 5) |
| --- | --- |

The leakage rate for the PEEK/PEEKsil leakage test systems can be calculated using the following equation,

| $leakage \left( \% \right)=\left( \frac{1}{1\text{+}\frac{R_{l}}{R_{main}}} \right)*100$ | (SE. 6) |
| --- | --- |

Note that equation SE. 6 implies that the leakage rate data collection can occur with variable flow rate as the leakage is independent of flow rate and only depends on the hydraulic resistance of the leakage and main channels, which based on equation SE 2 simplifies to the channel’s dimensions – length and diameter.

The details of the dimensions of the leakage and main channels including their internal diameter (ID) and length (L) are mentioned in the Table S1.

**Table S1.** Overview of the target leakages for PEEK and PEEKsil leakage test systems along with the internal diameter (ID) and length (L) of the leakage and main channels.

|  | | **leakage channel** | | **main channel** | |
| --- | --- | --- | --- | --- | --- |
|  | **leakage target %** | **ID (µm)** | **length (mm)** | **ID (µm)** | **length (mm)** |
| **PEEK** | 0.1 | 101.6 | 240 | 508 | 100 |
|  | 1 | 127 | 60 | 508 | 100 |
|  | 10.47 | 254 | 80 | 508 | 100 |
| **PEEKsil** | 0.09 | 50 | 50 | 300 | 50 |
|  | 1.38 | 100 | 50 | 300 | 50 |
|  | 11.47 | 175 | 50 | 300 | 50 |

To verify that our experiments were performed under laminar flow conditions we performed calculations of Reynolds number. The water $\rho$ (fluid/water density) is 1000 kg/m3 and μ (fluid/water viscosity) is 0.001 Pa.s. The internal diameters (D) were 508 µm and 300 µm for the PEEK and PEEKsil test setups, respectively. While we did not measure the flow rate, we can estimate based on the fluid volume used, and the time it took to use up the fluid volume, that our average flow rates (Q) were much less than 1000 µL/min which based on the SE.7 corresponds to the Re_max_= 41 and 70 for the IDs of the 508 and 300 µm, respectively, for PEEK and PEEKsil main channels, which is far less than the 2300 (critical Re). Further, Stokes flow, or creeping flow, occurs at lower Reynolds numbers < 1. Thus, we can assume that the flow is laminar.

| $Re=\frac{\rho\bar{v}D}{\mu}=\frac{4\rho Q}{\pi D\mu}$ | (SE. 7) |
| --- | --- |

Here, $\bar{v}$, $is the$ average velocity.

The schematic of the analytical model for the COC chips is shown in Figure S2.


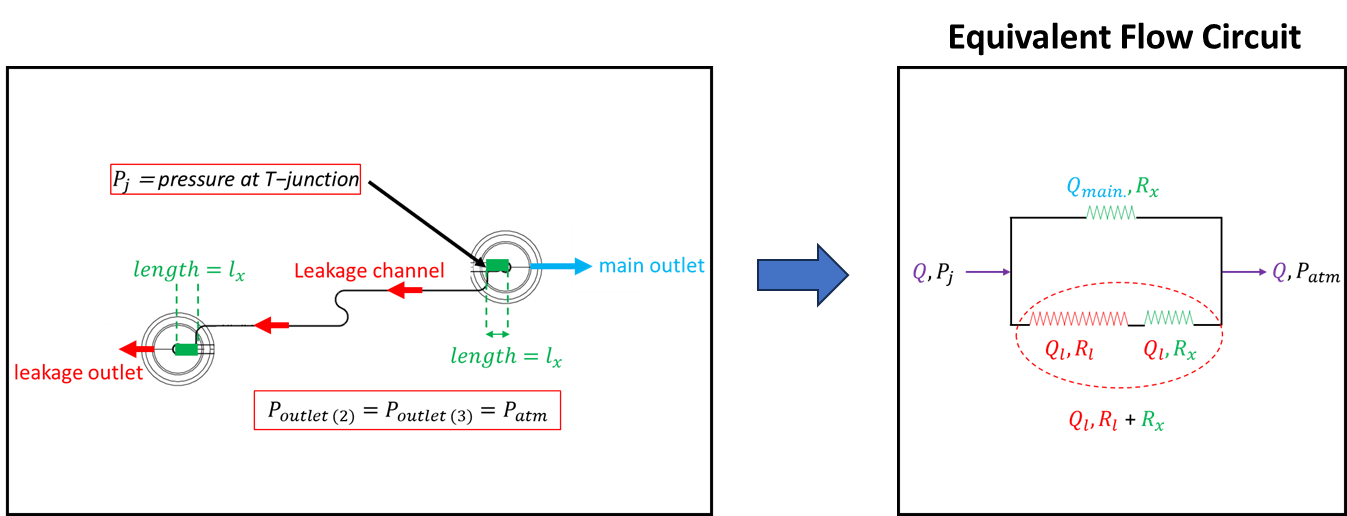


**Figure S2.** Schematic of the analytical model for the COC chip.

| and  $Q_{l}=\frac{P_{j}-P_{atm}}{R_{l}\text{ + }R_{x}}$  $Q_{main}=\frac{P_{j}-P_{atm}}{R_{x}}$ | (SE. 8) |
| --- | --- |

Q_main_ and Q_l_ are the flow rates of the main and leakage channel outlets, respectively. R_l_ and R_x_ are the hydrodynamic resistance of leakage channel and the part of main channel right after the junction, respectively. The P_j_ and P_atm_ are the pressures at the junction of main and leakage channels and the ambient pressure, respectively.

| $\frac{Q_{l}}{Q}=\frac{Q_{l}}{Q_{main}+Q_{l}}=\frac{R_{x}}{R_{l}\text{ +2 }R_{x}}=\left( \frac{1}{2\text{+}\frac{R_{l}}{R_{x}}} \right)$ | (SE. 9) |
| --- | --- |

And the leakage for the COC chips can be calculated using the following equation,

| $leakage \left( \% \right)=\left( \frac{1}{2\text{+}\frac{R_{l}}{R_{x}}} \right)*100$ | (SE. 10) |
| --- | --- |

The equations provided above have been coded into the attached Microsoft Excel^®^ application provided below. The developer should enter the length (L) and Internal Diameter (ID) of the fluidic elements (i.e. tubing and connectors) in it and the spreadsheet calculates the hydrodynamic resistance and then predicts the leakage percent using equation SE10. Note that this spreadsheet assumes that the connectors (e.g. junctions) and other elements used are same as what we used. Given that this is an open source spreadsheet, the developer can modify the other aspects of the spreadsheet as well.

**S2. Target Leakage and Obtained Leakage across three leakage test systems**

| (a) | (b) | (c) |
| --- | --- | --- |
| 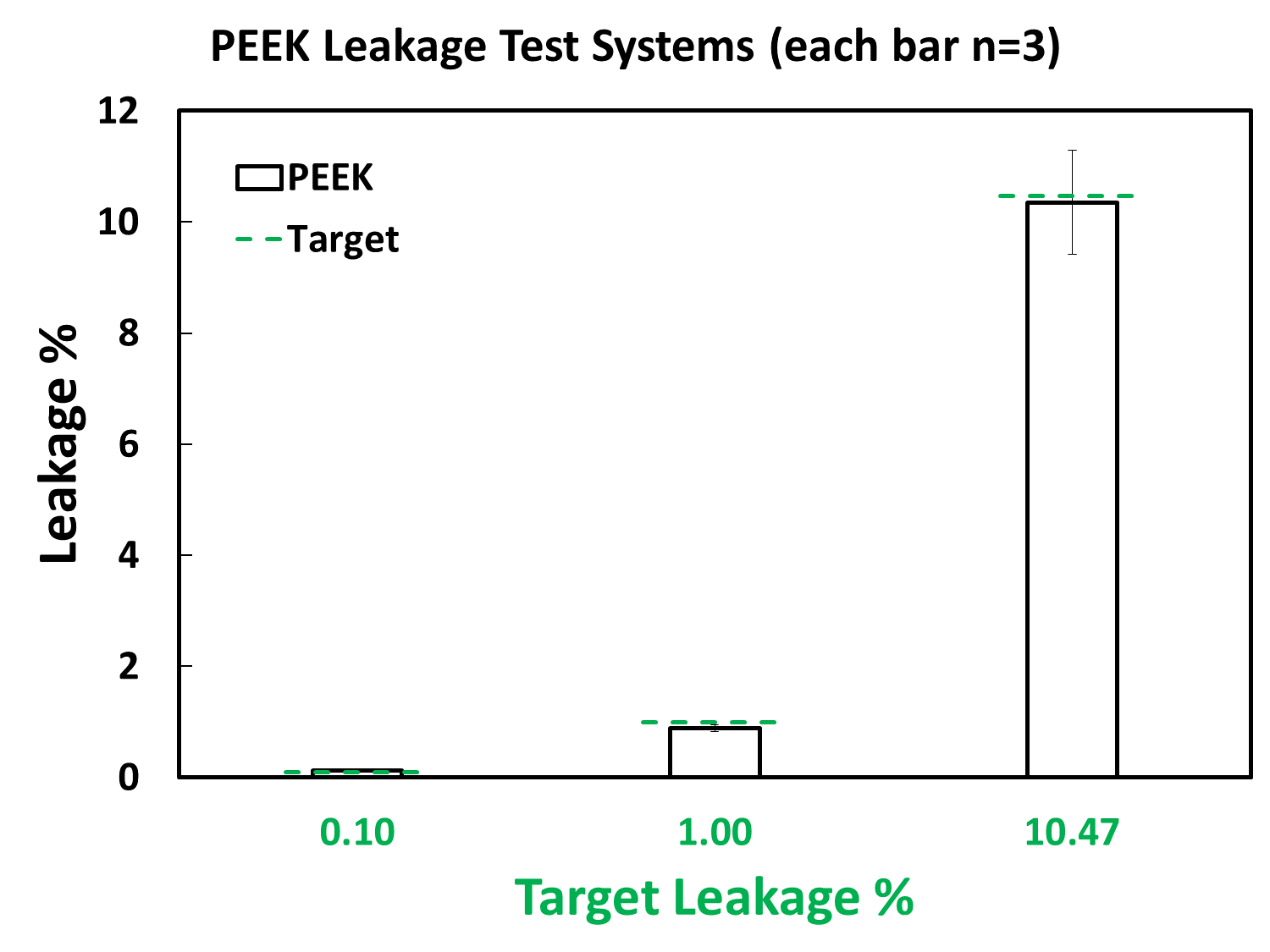 | 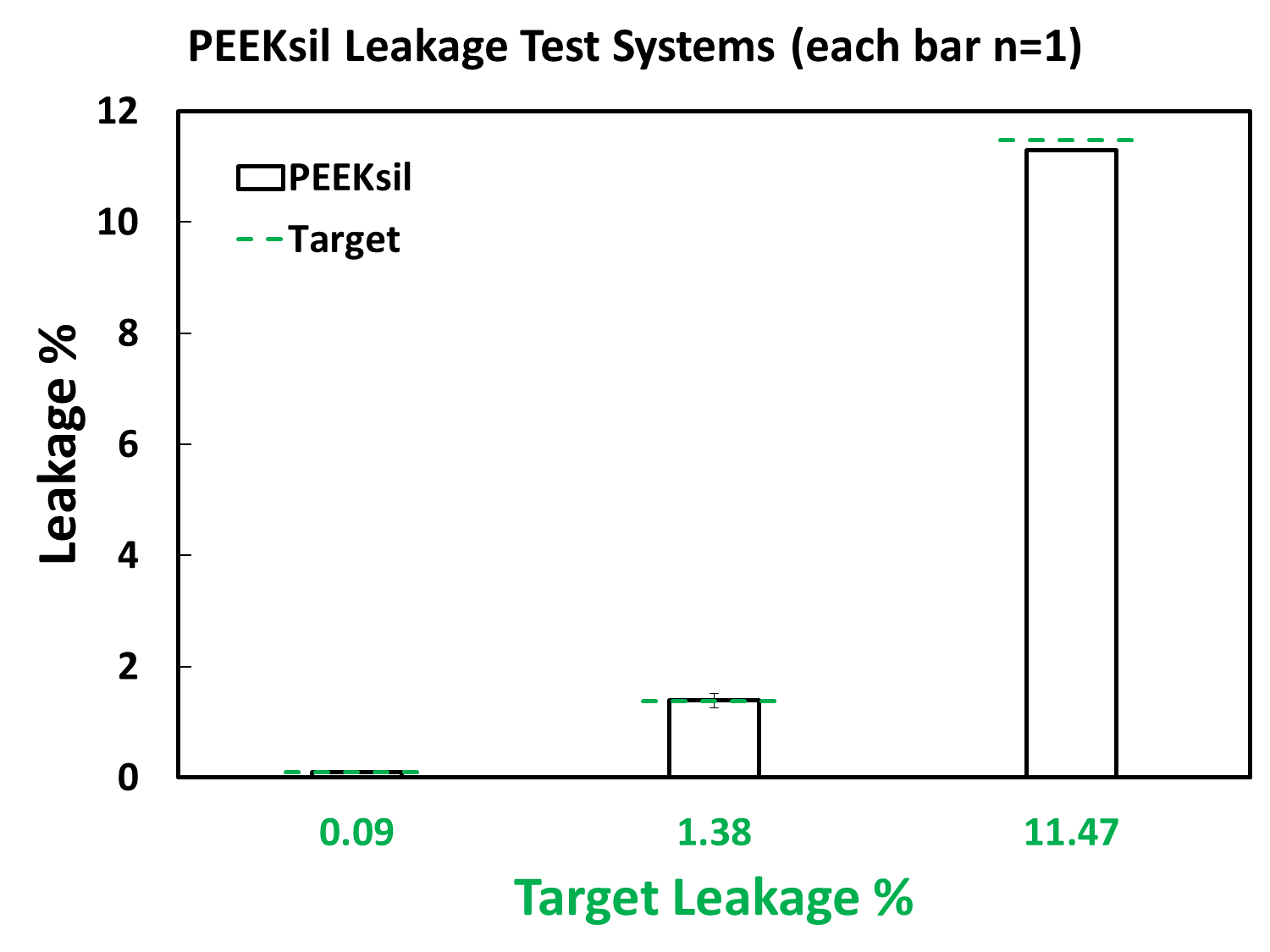 | 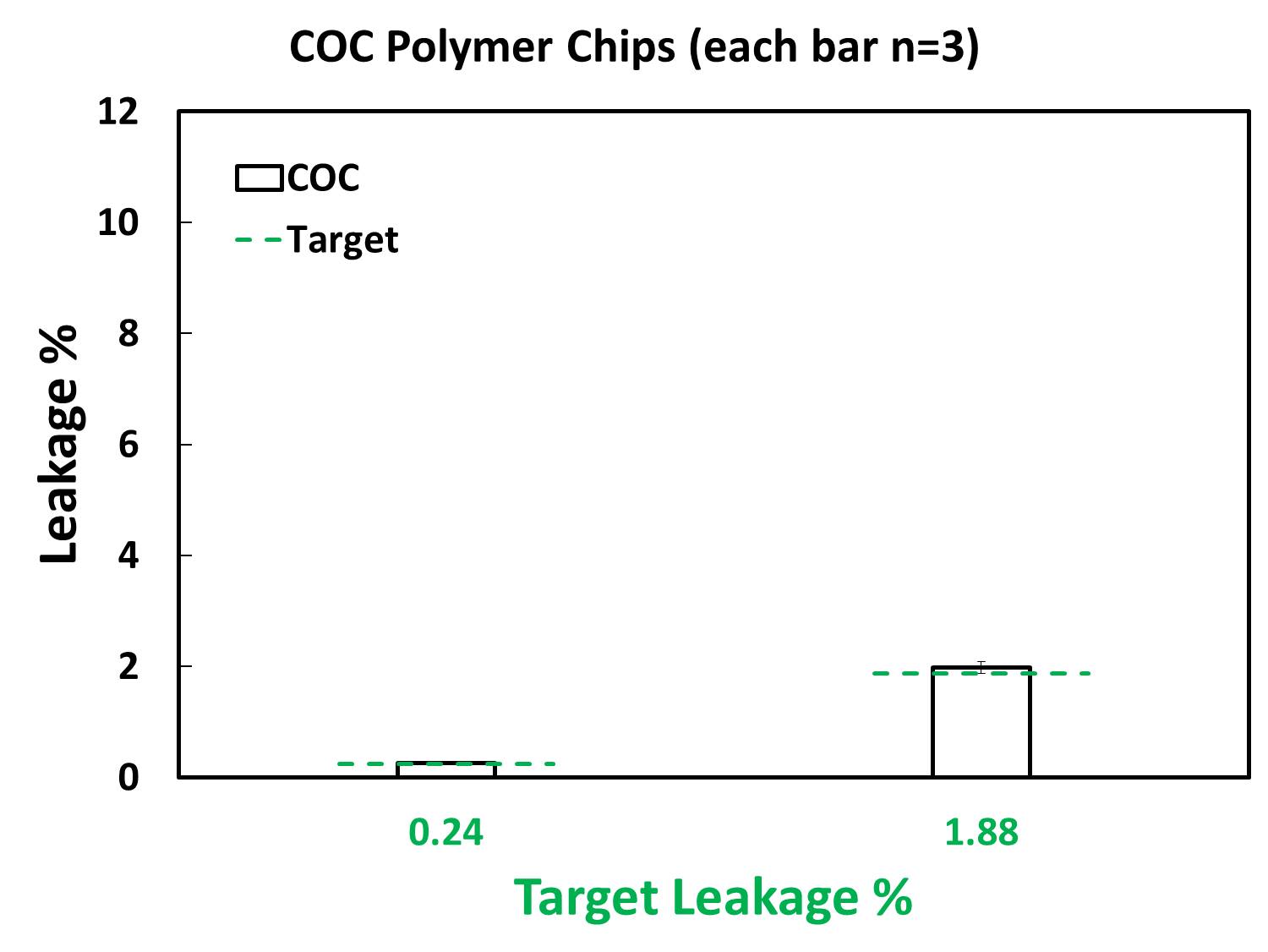 |

**Figure S3.** Average experimental and target leakages: (a) All PEEK leakage test systems, and each bar represents three leakage test systems tested in triplicate experiments (n=3). (b) All PEEKsil leakage test systems, and each bar is one leakage test system tested in triplicate experiments (n=3). (c) COC Polymer chips with 50*50 µm and 100*100 µm leakage channels, with target leakage percentages of 0.24 and 1.88, respectively, and each bar represents three leakage test systems tested in triplicate experiments (n=3).
